# Rare Association of Budd-Chiari Syndrome With Celiac Disease: A Case Report

**DOI:** 10.7759/cureus.11077

**Published:** 2020-10-21

**Authors:** Muhammad Saad Choudhry, Syed Muhammad Hussain Zaidi, Osama Mohiuddin, Anosh Aslam Khan, Amber Hanif

**Affiliations:** 1 General Surgery, Civil Hospital Karachi, Dow University of Health Sciences, Karachi, PAK; 2 Internal Medicine, Civil Hospital Karachi, Dow University of Health Sciences, Karachi, PAK

**Keywords:** celiac disease, budd-chiari syndrome, pakistan

## Abstract

Budd-Chiari associated with celiac disease is a rare phenomenon in the medical literature with annual incidence of less than five per million. The majority of the cases are reported from the North African region. Our patient presented in the out-patient department with symptoms of progressive abdominal distension, diffuse abdominal pain and shortness of breath for one year. She was a known case of celiac disease for the last three years. The clinical examination revealed ascites, jaundice, decreased air entry in basal segments bilaterally, and multiple hemangiomas all over the body. Haematological and biochemical investigations, including levels of pro-thrombotic factors and homocysteine level, turned out to be normal. However, computed tomography (CT) revealed hepatic vein obstruction. Hence, a diagnosis of Budd-Chiari syndrome was confirmed. The patient was managed with anticoagulants, diuretics and gluten-free diet. Within a month, the patient showed marked improvement with a significant reduction in ascites. To the best of our knowledge, this rare association is the first case to be reported from Pakistan and third from the region of South Asia.

## Introduction

Existing medical literature demonstrates that diseases frequently occur in association and often share common pathogenetic mechanisms. Herein, we report a rare association between Budd-Chiari syndrome (BCS) and celiac disease (CD). BCS, exceptionally rare itself, involves the obstruction of hepatic veins as a result of non-thrombotic or thrombotic occlusion (around 80% have an underlying hypercoagulable state) without the presence of right heart failure, sinusoidal obstruction syndrome or constrictive pericarditis [1]. The cardinal clinical features of BCS are hepatomegaly, ascites, and abdominal pain. A meta-analyisis by Li et al. reported an annual incidence of BCS to be 0.168-4.09 per million, whereas a subgroup meta-analysis revealed a cumulative annual incidence of 0.469 per million in Asia [2].

Celiac disease (CD), a rather common disorder, is an autoimmune condition involving the small intestine that occurs in genetically predisposed individuals. It results from the ingestion of gluten (a protein found in wheat, rye, and barley) [3]. According to a meta-analysis, the prevalence values for celiac disease were estimated to be 0.6% in Asia; higher in female vs male (0.6% vs 0.4%; P < 0.001) and greater in children vs adults (0.9% vs 0.5%; P < .001) [4]. CD has been reportedly associated with a range of extra-intestinal manifestations which also include several hepato-biliary disorders such as isolated hypertransaminasemia, autoimmune hepatitis, primary biliary cirrhosis, primary sclerosing cholangitis, and non-alcoholic fatty liver disease [5]. Furthermore, some cases of Budd-Chiari syndrome with chronic celiac disease have been reported from North Africa. Meanwhile, few cases have also been reported from other countries like Spain, Argentina, Turkey India and Czech Republic [1,6,7]. To the best of our knowledge, we present the first reported case of BCS with CD in Pakistan. 

## Case presentation

A 25-year old young female, with a known case of celiac disease for the last three years, presented in the out-patient department with primary complaints of progressive abdominal distension, abdominal pain and shortness of breath for one year. The abdominal distension started from the flank region bilaterally and then progressively became generalized. It was associated with abdominal pain which was constant, moderate in intensity, diffuse and dull in character. She also had a history of intermittent productive cough for past one year. It was associated with shortness of breath on exertion which had progressively increased and was now occurring at rest. According to the patient, she had several episodes of altered bowel movements and bloating for the past eight months which has increased in frequency over the time, as she has not been strictly adherent to a gluten-free diet over the last year. 

On admission, the patient was responsive and alert but appeared to be in distress. Her pulse was 95 beats per minute (BPM), blood pressure (BP) was 110/70 mmHg and respiratory rate was 23/minute. The general physical examination revealed pallor, jaundice, and peripheral cyanosis. There was also evidence of clubbing, bilateral peripheral edema, palmar erythema and multiple hemangiomas diffusely present all over the body. On abdominal examination, the abdomen was diffusely distended, tense, and non-tender. The fluid thrill was positive, however, the size of spleen and liver could not be appreciated because of massive ascites. Bowel sounds were normoactive. No hepatic or renal bruits were audible. Pulmonary auscultation revealed harsh vesicular breathing all over the chest with decreased air entry in basal segments bilaterally and occasional crepitations. There was dullness to percussion and decreased resonance in the left basilar chest region. Cardiovascular examination was unremarkable.

Hematological investigation displayed normocytic anemia (hemoglobin level of 9.9 g/dL), platelet count of 191000/mm3, and WBC count of 7200/µL. Liver function tests revealed albumin level of 2.8 g/dL (normal: 3.5-5.0 g/dL), total bilirubin of 5.9 mg/dL (normal: 0.1-1.2 mg/dL), alkaline phosphatase of 674 U/L (normal: 0-240 U/L), and gamma-glutamyl transpeptidase of 65 U/L (normal: 6-42 U/L). Pro-thrombin (PT) time and international normalized ratio (INR) were elevated at 20 seconds and 1.93 seconds, respectively, while partial thromboplastin time was within normal limit. Serum iron levels were 33 mcg/dL (normal: 60-170 mcg/dL), while ferritin level was normal. Vitamin B12 level was at 163.8 pg/mL (normal: 208-964 pg/mL) and folate level was normal. Vitamin D (25-OH) was decreased at 12.89 ng/mL (normal: >29 ng/mL). Serum calcium levels were within normal limits. A complete serological screen for autoimmune hepatitis (anti-nuclear antibodies and anti-smooth muscle antibodies) produced normal results (antibodies were undetected). Hepatitis C antibodies and HBsAg were both non-reactive. Moreover, serum ceruloplasmin levels were also within the normal range. Fasting lipid profile, kidney, and thyroid function tests were also within normal limits. A full screen for all known thrombophilic factors turned out to be insignificant. Specifically, the homocysteine level was 9.7 µmol/L (normal: 4.9-11.6 µmol/L ) while anticardiolipin antibodies (IgG and IgA), lupus anticoagulant and Factor V Leiden were all negative. Levels of antithrombin III, protein C, protein S were also within reference range. The ascitic fluid analysis revealed total protein 1.3 g/dL, albumin 1g/dL, glucose 95 mg/dL and serum-ascitic albumin gradient of 1.8, features suggestive of portal hypertension. A duodenal biopsy performed via upper gastrointestinal endoscopy gave evidence of villous atrophy, crypt hyperplasia, and markedly increased intraepithelial lymphocyte (Marsh grade III). Furthermore, anti-tissue transglutaminase IgA levels were 154.6 IU/ml.

**Figure 1:**
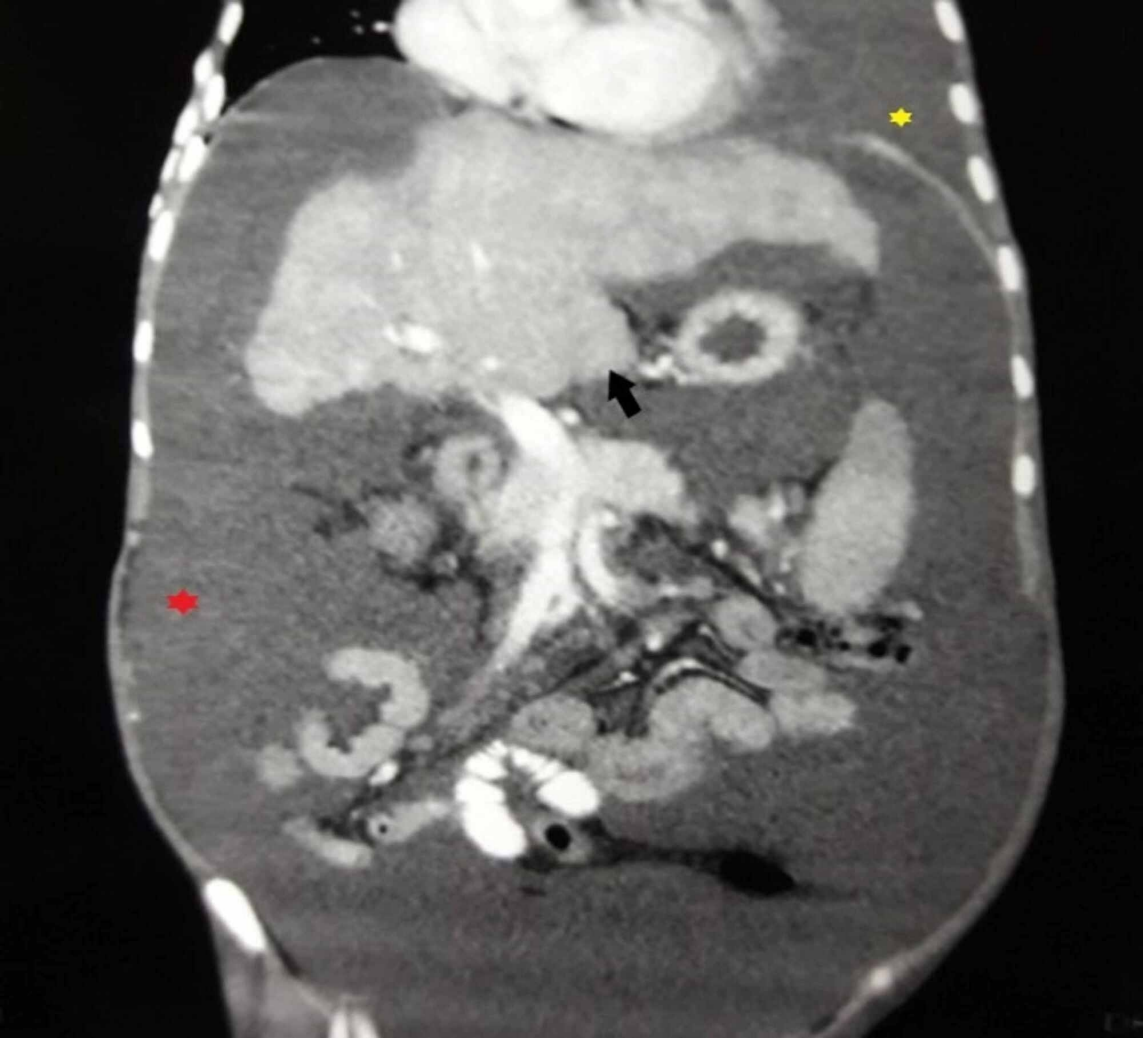
Coronal contrast computed tomography (CT) obtained during venous portal phase shows altered liver parenchyma with hypertrophy of caudate lobe (black arrow). Left pleural effusion (yellow *) and massive ascites (red *) can also be seen.

**Figure 2:**
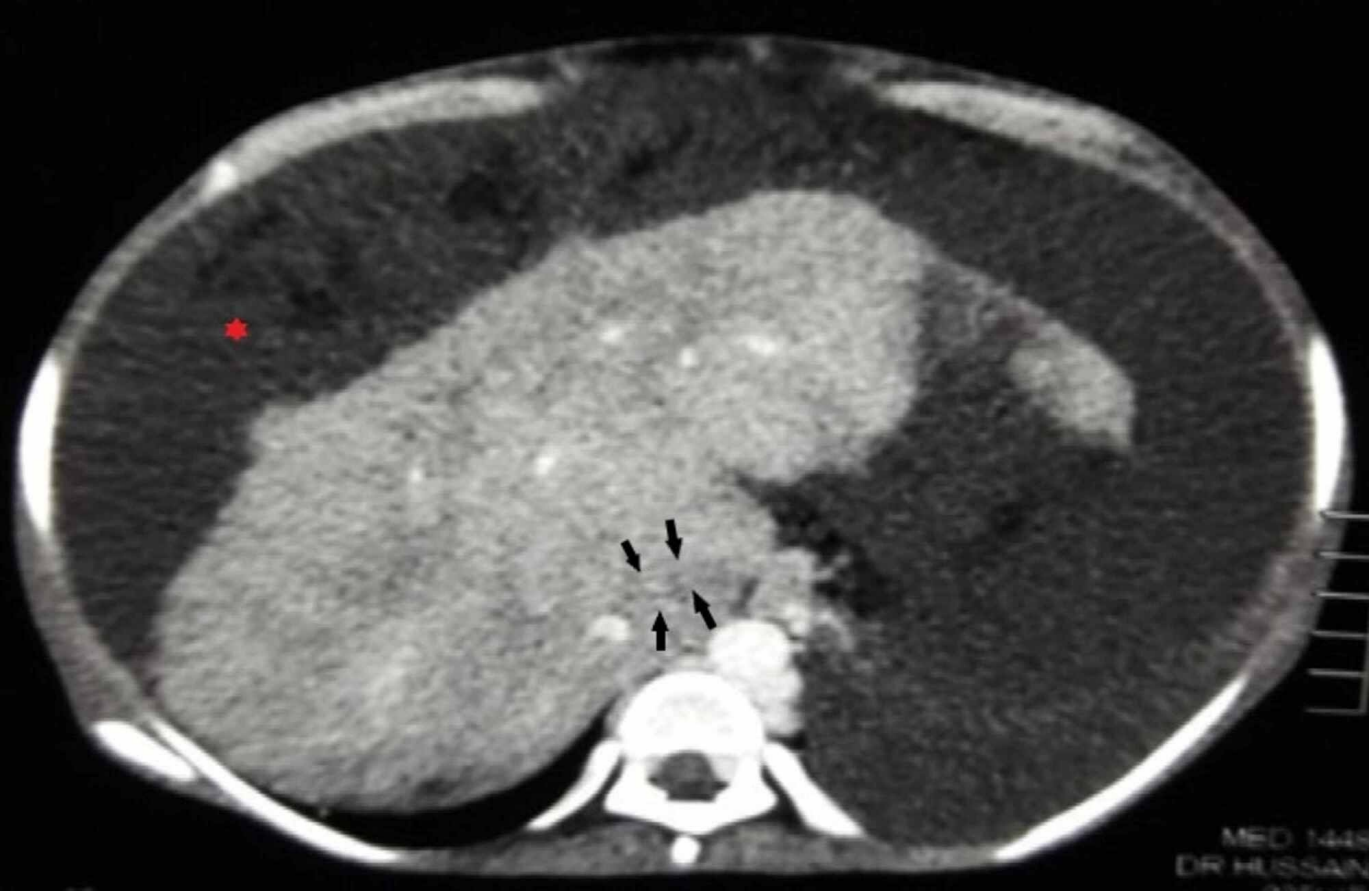
Axial contrast computed tomography (CT) obtained during venous portal phase shows non-opacification of hepatic vein (black arrow) with evidence of ascites (*).

Thereafter, an abdominal ultrasound was performed which showed a liver span of 8.5 cm (normal: 10 cm-12 cm), small in size with coarse echotexture and irregular margins, no focal mass, and intrahepatic duct dilatation. The splenic span was 14.5 cm (normal: 10.5 cm-12 cm) and splenic vein measured 1.2 cm. On Doppler ultrasonography, portal vein measured 0.7 cm with the normal hepatoportal monophasic flow. The velocity of the portal vein was 16 cm/sec (normal: 20 cm/sec-40 cm/sec), and all hepatic veins were attenuated. Subsequently, a contrast computed tomography (CT) of the abdomen was pursued (Figures 1-2) which displayed a cirrhotic liver with massive ascites and left-sided pleural effusion. No aneurysm, superior and inferior vena cava thrombosis or liver masses were seen. Caudate lobe hypertrophy and non-visualization of the hepatic vein confirmed the diagnosis of Budd-Chiari Syndrome.

The patient was managed conservatively with anticoagulants, diuretics, and a gluten-free diet in addition to oral vitamin D supplements and vitamin B12 injections. Within four weeks, there was a marked decrease in ascites with improvement in liver functions (total bilirubin was 1.9 mg/dL and albumin 3.8 g/dL).

## Discussion

Our case demonstrates Budd-Chiari syndrome (BCS) in a female patient previously diagnosed with celiac disease, in the absence of a pro-thrombotic state. The patient was not adherent to a gluten-free diet for several months before her diagnosis of BCS. This rare association was first reported in 1990 [8]. Studies have shown that various extraintestinal disorders have been associated with CD including type 1 diabetes mellitus, dermatitis herpetiformis and autoimmune thyroiditis. Other hepato-biliary disorders like mild isolated hypertransaminasemia, autoimmune hepatitis, primary biliary cirrhosis, and primary sclerosing cholangitis have also been reported [5]. BCS associated with CD is more prevalent in North African countries relative to our region of Southeast Asia, possibly because of the high prevalence of the celiac disease in North Africa [9].

One multicentric case series showed that the majority of patients reported belonged to the female gender and presented in the third or fourth decade [1]. Likewise, our patient was also a female in her second decade of life. Our patient presented with abdominal pain and distension that progressively increased over a period of one year causing significant discomfort. It can be attributed to the development of large collaterals secondary to chronic hepatic vein thrombosis. Therefore, physicians should consider the possibility of incidental finding of BCS in CD patients with unexplained ascites, right upper quadrant abdominal pain, jaundice, or hepatomegaly [10]. Clinical presentation of Budd-Chiari syndrome is reported to range between fulminant and chronic, with more frequent presentation of the latter. The case described herein had a similar course of chronic presentation, as demonstrated by imaging modalities and histopathology. 

In Budd-Chiari syndrome, an underlying prothrombotic state can be detected in 80% of patients. On the other hand, when seen in association with CD, no specific thrombotic etiology was identified in most cases, which is similar to our case presentation. [11]. Some of the possible mechanisms that may cause underlying hypercoagulable state include myeloproliferative disorders, autoimmune vasculitis, antithrombin III deficiency, factor V Leiden mutation and reduced vitamin K absorption leading to decreased protein C and S [12]. Folate deficiency or methylenetetrahydrofolate reductase (MTHFR) gene mutation has been attributed to the development of hyperhomocysteinemia, which may lead to thrombosis in Budd-Chiari syndrome [13]. In our patient, we did not find any of these factors responsible for thrombosis. However, investigation for MTHFR gene mutation could not be performed due to financial limitations. Since there is a prevalence of non-thrombotic etiology in the vast majority of BCS associated with CD, other triggering mechanisms like genetics, diet and environment should also be studied in depth [1].

## Conclusions

Our case report features the rare association between Budd-Chiari syndrome and celiac disease. Although a limited number of cases have been reported in the medical literature so far, physicians should keep a high index of suspicion. We propose that the diagnosis of Budd-Chiari syndrome should be taken into consideration when patients with celiac disease present with signs and symptoms of liver injury. Further research needs to be done on pathological mechanisms leading to the development of such an association and the role of a gluten-free diet on the clinical course of the disease. Several etiological factors, including prothrombotic, non-thrombotic environmental, genetic and dietary, need to be investigated thoroughly to identify the triggering mechanism.
